# Nanotechnology in food systems: opportunities and risks for human health

**DOI:** 10.5114/bta/209978

**Published:** 2025-12-06

**Authors:** Sonam Yadav, Jaishiv, Rohit Kumar

**Affiliations:** 1 Indian Pharmacopoeia Commission, Ministry of Health & Family Welfare, Government of India, Raj Nagar, Ghaziabad, Uttar Pradesh, India; 2 Department of Chemistry, IFTM University, Moradabad, Uttar Pradesh, India

**Keywords:** nanotechnology, food systems, nanomaterials, nanoparticles, health, risk assessment

## Abstract

Nanotechnology has emerged as a promising field with the potential to revolutionize several industries, including the food industry. It offers innovative solutions to critical challenges in food, such as safety, nutrition, waste reduction, and sustainability. This study examines the possibilities offered by nanotechnology in the food sector, with a focus on risk assessment, safety evaluation, and regulatory approaches. While nanotechnology in food applications presents many advantages, it also raises concerns about potential health risks. Due to their distinct characteristics, nanoparticles may interact with living organisms in unpredictable ways, creating challenges for risk assessment and management. This review also explores the possible hazards of using nanomaterials in the food system, highlighting the need for comprehensive toxicity studies and effective regulatory frameworks. Addressing these issues requires a multidisciplinary approach involving collaboration among scientists, regulators, policy-makers, and stakeholders to balance the benefits and risks of nanotechnology in the food system. As the food sector seeks novel approaches to meet rising global demand, it is crucial to thoroughly assess both the advantages and risks of nanotechnology to ensure its responsible and sustainable application while protecting human health and the environment.

## Introduction

Nanotechnology is one of the most promising fields for enhancing food availability and developing innovative products across diverse sectors, including food, water, agriculture, the environment, medicine, energy, and electronics (Sadeghi et al. 2017). By enabling the precise manipulation of atoms and molecules at their most fundamental level, nanotechnology has led to the creation of novel materials with exceptional properties. The term *nano* originates from the word *dwarf*, signifying something extremely small – one billionth of a meter (10^-9^), or approximately one nanometer (nm). This scale is roughly 3–5 atoms wide and about 40,000 times thinner than a human hair or comparable to the thickness of a virus (100 nm). Nanotechnology primarily focuses on creating materials ranging from 1 to 100 nm in size (Ullah et al. 2024). In food science, this technology is applied to develop materials with enhanced stability, solubility, and bioavailability (Chudasama and Goyary 2024). Scientists and industry experts have already recognized the vast potential of nanotechnology in nearly every aspect of the food industry – from agriculture and food processing to packaging, safety, and nutrient delivery – promising transformative advancements in these areas (Pathakoti et al. 2017).

Nanotechnology bridges the gap between conventional and quantum mechanics through the utilization of mesoscopic systems – an intermediate domain. In medicine, these mesoscopic systems enable the development of nano-assemblies, including agricultural products, nanomedicine, and nanotools designed to enhance therapies and diagnostics. Nanomaterials (NMs) are being widely applied in regenerative medicine, advancing fields such as tissue engineering, cell therapy, and gene sequencing. Research has documented nano-assemblies with properties that support cell adhesion, migration, and differentiation. Nanotechnology is also making notable contributions to microbiology and antiviral research, offering innovative solutions for disease prevention and treatment (Malik et al. 2023).

On September 9, 2003, the United States Department of Agriculture (USDA) released its first roadmap for integrating nanotechnology into the food industry. As a partner agency in the Federal National Nanotechnology Initiative (NNI), the USDA had previously organized a National Planning Workshop, *Nanoscale Science and Engineering for Agriculture and Food Systems*, in November 2002 in Washington, DC. The workshop explored the potential of nanotechnology in transforming agriculture and food systems and identified key opportunities for its application. Its outcome was the development of a scientific roadmap and strategic plan, which included recommendations for launching a dedicated nanotechnology program within the USDA to advance agriculture and food systems (Siddiqui and Alrumman 2021; U.S. Department of Agriculture 2003).

Over the past few decades, nanotechnology has increasingly become a part of daily life through applications in electronic chips, textiles, paints, agriculture, food processing, packaging, wastewater treatment, clinical diagnostics, therapies, and environmental restoration (El-Sheekh et al. 2022). In recent years, substantial evidence from industrialized countries suggests that nanotechnology holds considerable potential for addressing environmental challenges. Its applications include improving drinking water hygiene, detoxifying pollutants such as toxic heavy metals and organochlorine pesticide residues, and reprocessing these materials through advanced techniques like nanofiltration (Taran et al. 2021).

Nanopesticides have been ranked first among the ten chemical innovations identified by the International Union of Pure and Applied Chemistry (IUPAC) as having the greatest potential to shape the future of human civilization. This recognition stems from their ability to minimize negative impacts on the environment and public health while providing effective pest management solutions (Gomollón-Bel 2019). In agriculture, pesticides incorporating nanotechnology benefit from improved penetration, coverage, and absorption facilitated by NMs. The delivery of nano-agrochemicals through NMs holds significant potential to enhance food security while promoting the ecological sustainability of agricultural practices. This is achieved by reducing labor costs, minimizing environmental pollution, and increasing the efficiency of agricultural inputs (Li et al. 2021; Su et al. 2020; An et al. 2022). In the food sector, nanotechnology also contributes to food safety through nanosensors that detect contamination during production, processing, storage, packaging, and transportation (Nile and Kai 2021). Moreover, nanotechnology-enabled processing and packaging have proven highly effective in improving both the efficiency and safety of the food system (Weiss et al. 2006).

Nanotechnology has made significant contributions to food science in several key areas, including enhancing the tracking and tracing of pollutants, extending the shelf life of food products, improving storage, and enabling the incorporation of antibacterial agents and health supplements (Neo et al. 2013). Researchers have developed numerous technologies to improve food quality and safety, with nanotechnology playing a pivotal role in producing food with higher oral bioavailability, improved solubility, and enhanced thermal stability (Semo et al. 2007).

The application of nanotechnology in packaging is often classified based on functionality. Most NPs used in food packaging exhibit antimicrobial properties, act as carriers for antimicrobial polypeptides, and protect against microbial spoilage (Nile et al. 2020). Nanostructures introduced into the food industry serve two major purposes: food ingredients and sensors. Nano-food ingredients have diverse applications in food processing and packaging, offering functionalities such as antimicrobial and anticaking agents, nano additives, nanocarriers, and nanocomposites. Additionally, nanosensors are incorporated into food packaging to monitor and ensure food quality throughout storage and distribution (Sahani and Sharma 2021).

A new revolution is underway in the Agri-tech sector, aiming to sustainably meet the growing global food demand. Advances in nanotechnology have led to increased use of NMs, highlighting their potential to address these challenges effectively (Zain et al. 2023). Nanotechnology is a rapidly expanding field with applications spanning energy, medicine, food, and many other industries (Arpanaei et al. 2024). The market for nanotechnology-enabled products has shown steady growth, currently generating over $1 billion annually. By 2024, it is projected to surpass $125 billion, driven by advancements across electronics, pharmaceuticals, automotive, agriculture, and other sectors (Santos et al. 2024).

**Figure 1:**
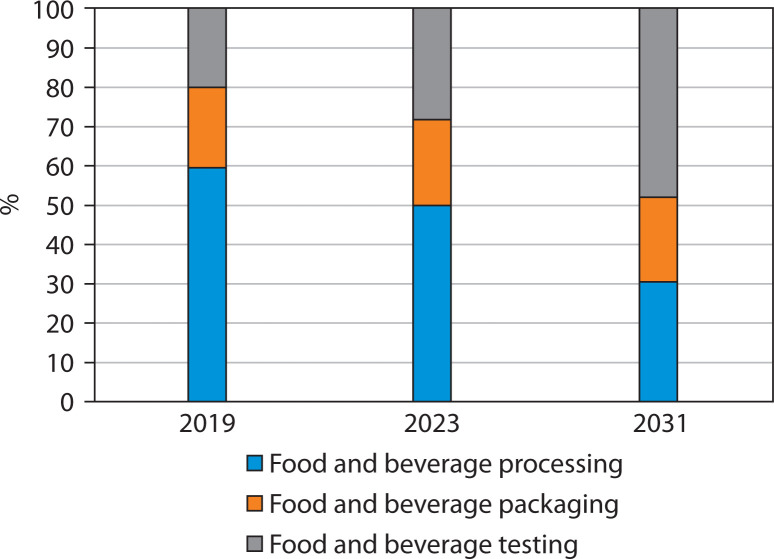
Adoption trends of food nanotechnology: market share (%) by application (2019–2031)

The presence of more than a thousand NMs-containing products on the market underscores nanotechnology’s status as a rapidly growing multibillion-dollar industry. In the past decade, over 300 nanofood products have been introduced to international markets, reflecting its increasing influence in the global food sector. Figure 1 illustrates *Adoption trends of food nanotechnology: market share (%) by application (2019–2031)* (Mali 2023).

## Opportunities in food systems

There are many advantages to employing nanoparticles (NPs) in the packaging sector, as mentioned above. However, the increased toxicity associated with the behavior of particles on the nanometric scale raises serious concerns about the use of these nanostructures. These particles have the potential to harm consumers when they migrate from food packaging into food. When creating any novel food packaging material, it is necessary to examine the component migration behaviors to see if any unwanted or dangerous components are leaking into the food (Gupta et al. 2023).

The European Food Safety Authority’s (EFSA) guidelines, the amount of silver that migrates from packaging materials into food must not exceed 0.05 mg/kg in food and 0.05 mg/l in water. The US Environmental Protection Agency states that silver levels in drinking water should not be higher than 0.10 mg/l. A disadvantage of silver is its potential to migrate into food products, thereby increasing the risk of toxicity, even though it can extend the shelf life of food products (Maria et al. 2024).

Despite growing agricultural challenges, combining nanotechnology with conventional methods offers a forward-thinking approach to ensuring food security. The potential of nanotechnology to enhance food security, identify pathogens, treat illnesses, enable effective delivery systems, and produce diverse packaging materials is considerable (Saha et al. 2024).

Recent developments in nanotechnology and biotechnology, such as precision fermentation, are examples of biological technologies. These technologies use biological processes and organisms to produce food ingredients or improve the safety and quality of food. They provide innovative ways to enhance nutritional value, extend shelf life, and promote sustainable food production practices. However, they also raise issues related to consumer acceptance, legal challenges, and the ethics of altering living organisms (Hassoun et al. 2024).

NPs can readily cross tissue and cell barriers due to their small size and large surface area, which may harm biological systems. In addition to an NPs size and shape, its charge and digestibility can also influence its potential toxicity. Numerous studies have shown that nanoemulsions have higher bioavailability, which may cause adverse health effects in some populations when consumed in excess for certain substances. The type and quantity of ingredients used to create nanoemulsions represent another potential source of toxicity. In some cases, small droplet sizes can only be achieved using small-molecule synthetic surfactants, which may be more toxic than natural emulsifiers, and higher levels of emulsifiers are required to stabilize their larger surface areas (Kaur et al. 2024).

Additionally, surface coatings such as polyethylene glycol (PEG) can alter cellular uptake, reduce immunological recognition, and improve biocompatibility and systemic circulation. Because poorly degradable NPs can accumulate and have long-term toxicity, digestibility and biodegradability are also important considerations. These elements influence the biodistribution, cellular interaction, and toxic potential of NPs (Nel et al. 2006). Additionally, the way that NPs interact with cellular membranes and how toxic they are overall are greatly influenced by their surface charge. Stronger electrostatic interactions between positively charged NPs and the negatively charged phospholipid bilayer of cell membranes can result in increased cytotoxicity and membrane disruption as well as improved cellular uptake. Conversely, neutral or negatively charged NPs may be absorbed less effectively but are typically less harmful. For instance, a study showed that, in contrast to their anionic or neutral counterparts, cationic gold NPs caused increased levels of reactive oxygen species (ROS) and cell membrane damage in HeLa cells. Similarly, a study showed that surface charge influences the overall biocompatibility of the NMs by influencing intracellular localization, immune response activation, and uptake efficiency (Zhao et al. 2011; Fröhlich 2012).

The food industries are looking for innovations in packaging unit operations in this age of technological advancements in every sector because of the many detrimental effects of traditional packaging materials, which are constantly contaminating the environment. In order to enhance the physicochemical and functional qualities of packaging film, the food packaging industry is currently utilizing a number of NMs in the food packaging system (Gupta et al. 2024).

## Types of NMs used in food

There are many uses for nanotechnology in the food sector. In these applications, a particular food product is combined with a specific type of NM to provide desirable properties (Bajpai et al. 2018). Table 1 presents the applications and mechanisms of NMs in the food sector (Nile et al. 2020). NMs are defined as materials with sizes ranging from 1 to 100 nm (Dazon et al. 2020). They have developed into a flexible platform capable of offering economical, environmentally friendly, and efficient solutions to global challenges (Singh et al. 2023).

**Table 1:** Applications and mechanisms of nanomaterials in the food sector

S no.	Nanomaterial source	Category	Mechanism	Technology	Applications
1	Iron	Inorganic nanoparticles	Enhanced bioavailability, improved absorption, controlled release, reduced toxicity, immune function enhancement, and antioxidant effects	Chemical reduction, solgel synthesis, green synthesis, spray drying, coprecipitation, and encapsulation technology	Food supplement
2	Silver		Antimicrobial action, anti-inflammatory effects, bioavailability in food supplements, surface disinfection, and synergistic effects	Chemical reduction, biosynthesis, physical vapor deposition, electrochemical synthesis, encapsulation, and spray coating	Food supplements, antimicrobial agent – used in food contact surfaces (cutlery, storage containers, fridges, and worktops)
3	Iridium		Enzyme mimicry, antioxidant properties, cellular protection, anti-inflammatory effects, detoxification, and biocompatibility and uptake	Chemical reduction, biosynthesis, physical vapor deposition, encapsulation, spray drying, and functionalized nanoparticles	Food supplement
4	Platinum		Enzyme mimicry, antioxidant activity, anti-inflammatory effects, detoxification, cellular uptake, and stabilization of nutrients	Chemical reduction, green synthesis, physical vapor deposition, encapsulation, spray drying, and nano-functionalization	Food supplement
5	Zinc		Enhanced bioavailability, targeted nutrient delivery, improved stability, antioxidant properties, immunomodulation, and coloring agent mechanism	Sol-gel synthesis, biosynthesis, physical vapor deposition, nano-chelation, spray drying, and liposomal encapsulation	Food supplement/colorant
6	Liposomes	Organic nanoparticles	Encapsulation, protection, controlled release, targeted delivery, and enhanced bioavailability	Thin-film hydration, ultrasonication, microfluidization, reverse phase evaporation, supercritical fluid technology, and spray drying	Encapsulation and targeted delivery of food components
7	Protein		Re-micellisation process, gelation properties, heat stability, improved emulsification, enhanced solubility, biocompatibility, and controlled release	Ultrasonication, high-pressure homogenization, electrostatic assembly, freeze-drying (lyophilization), enzymatic hydrolysis, spray drying, and controlled pH and ionic strength	Re-micellised calcium caseinate from dairy protein. Increased functionality (gelation, heat stability, and other properties)
8	Polymeric		Stability and encapsulation, release mechanism, surface modification, bioinertness, biodegradation, controlled release, and biocompatibility	Emulsion-solvent evaporation, nanoprecipitation, electrostatic assembly, coacervation, spray drying, and supercritical fluid technology	Non-degradable: polystyrene Biodegradable: PGLA, gelatin, and collagen
9	Globular proteins	Nanofibers/fibrils	Structural properties, thermal stability, increased shelf-life, gel formation, and increased functional properties	Nanoprecipitation, high-pressure homogenization, electrospraying, spray drying, and solvent evaporation	Thermal stability, increased shelflife. Formation of a transparent gel network for use as a thickening agent
10	Oil in water	Nanoemulsions	Formation and stabilization, delivery of active compounds, extended shelf-life, flavour release, and low-fat products	High-pressure homogenization, ultrasonication, phase inversion temperature, microfluidization, and solvent emulsification	Stabilization of biologically active ingredients, delivery of active compounds; extended shelf-life, flavor release, and low fat products
11	Calcium carbonate	Nanodispersions	Nanoscale size and surface area, enhanced solubility, stabilization in aqueous solutions, increased bioavailability and reduced grittiness, and sensory impact	High-pressure homogenization, ball milling, solvent evaporation, precipitation methods, and microfluidization	Increased solubility of calcium carbonate – can be used at higher addition levels
12	Clay composites	Nanoclays	Intercalation and exfoliation, improved barrier properties, enhanced mechanical strength, thermal stability and durability, and light and UV protection	Melt blending, solution casting, in-situ polymerization, electrospinning, and solvent casting	Used in packaging materials to extend shelf-life, durability, and thermal properties (includes nanolaminates)

NMs are grouped based on their size, properties, and structure. NMs with a high surface-to-volume ratio are particularly desirable for their physicochemical properties, such as bioavailability, diffusivity, solubility, optics, magnetism, strength, color, toxicity, and thermo-dynamics (Sahoo et al. 2021). Because of their superior physicochemical properties and antimicrobial activity, NMs are widely employed in water treatment, agriculture, healthcare, food safety, and preservation, as well as against a variety of pathogenic microbes (Baranwal et al. 2018). In addition to their unique physicochemical characteristics, NMs are also easily conjugable (Hu et al. 2020).

One general way to classify engineered NMs used in feed, food, and agriculture is as follows: inorganic (e.g., metal and metal oxide NPs), organic (mostly natural product NPs), or combination (e.g., surface-modified clays) (Peters et al. 2016). Inorganic NMs include metals, metal oxides, salts, and carbon-based materials such as carbon nanotubes, fullerenes, carbon black, and clay (King et al. 2018). Among these, silver NPs are the most widely produced and commercially used due to their antimicrobial activity, whereas gold (Au) NPs are extensively studied for use as sensors and detectors. Titanium dioxide (TiO_2_) NPs have also been investigated as a food preservative, primarily as a white pigment and taste enhancer (He et al. 2019).

Nanocomposites based on metals and metal oxides are applied in food coating and packaging. Silver nanocomposites and NPs are among the most widely used antimicrobials in the food industry (He and Hwang 2016). TiO_2_ can be incorporated into packaging materials as a coating agent to reduce *Escherichia coli* contamination (Chellaram et al. 2014). The use of NMs in food packaging is expected to reduce challenges associated with traditional packaging materials while lowering waste and conserving valuable raw ingredients (Sozer and Kokini 2009).

Numerous NMs are being developed as functional additives for food packaging, including nano-TiO_2_, silver NPs, nanoclay, titanium nitride NPs, and nano-zinc oxide (ZnO) (Pal 2017). To create biosensors for quantification of microbes and other testing for applications related to food safety, NMs such as metal NPs, carbon nanotubes, quantum dots, and other active NPs can be used (Inbaraj and Chen 2016).

**Figure 2:**
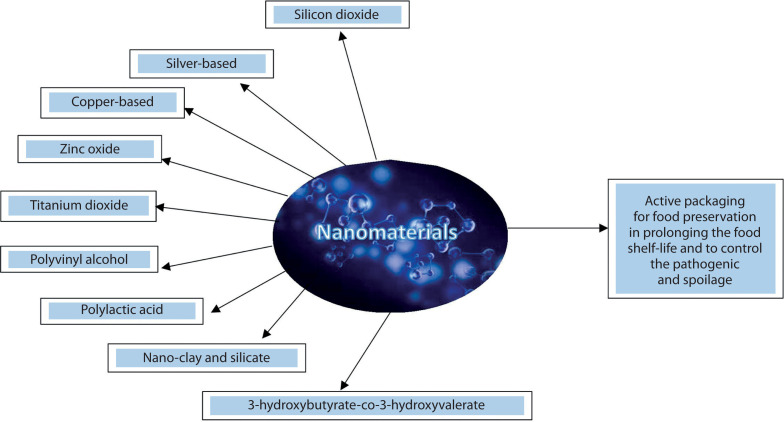
Types of nanomaterials used in food packaging

By construction, NMs are typically divided into five categories: metal-based, carbon-based, dendrimers, ceramics, and composites (Biswas et al. 2023). TiO_2_ is also widely used as a food additive in cake icing, puddings, candy, gum, and white sauces (Kumar et al. 2020). Figure 2 illustrates the types of NMs used in food packaging.

## Risks to human health toxicity

Nanotoxicity, or the harmful effects of NPs on biological systems, can involve several undesirable physiological reactions, such as internal and external interactions with NPs that cause cell disruption. A comprehensive understanding of NMs and the associated risk factors, including the physiological mechanisms of toxicity, plays a pivotal role in their future applications (Sharma et al. 2024). Several NMs have been reported to negatively impact the environment and human health. Table 2 presents various NMs and their toxicity mechanisms with corresponding physiological responses.

**Table 2:** Nanomaterials and their toxicity mechanism with physiological response

Nanomaterial	Toxicity mechanism	Physiological response	References
Iron oxide, gold	Internalization and membrane disruption. Highest cellular uptake with the least membrane disruption among all shapes, thus the least shape-dependent toxicity	Cell division dysfunction and disturbed cellular trafficking; Mechanical interference with the mitotic spindle and DNA	Hartig et al. (2007), Schaffazick et al. (2003), Lee et al. (2007)
SWCNT, MWCNT, gold, mesoporous-silica	Internalization and membrane disruption. Severe influence on the initiation of phagocytosis. Blockage of transport channels. The highest distorting force on the cell membrane among all shapes. Smaller aspect ratios lead to faster internalization and less cell membrane disruption	Chronic inflammation due to frustrated phagocytosis, mutagenic events, and mesothelioma formation	Aaron et al. (2011), Alkilany et al. (2010), Andelman et al. (2010), Hsiao and Huang (2011), Peng et al. (2011), Sun et al. (2011), Yang and Cui (2008), Duan et al. (2018)
Gold	Dependent on the average radius of curvature. Disruption of membrane integrity and transport may occur	Toxicity due to chronic inflammation or impaired phagocytosis	Chithrani et al. (2006)
Nickel, carbon black	Aggregation or agglomeration changes the size of particles, thus increasing their visibility to macrophages	Aggregation changes the retention time of particles; changes in size may increase or decrease toxicity	Sager et al. (2007)
ZnO, iron oxide	Aggregation and cell membrane disruption may be dependent on the prevalence of high aspect ratio particles	Combinational effect similar to aggregated particles and fibrous particles	Mercer et al. (2008), Vevers and Jha (2008)
Quantum dots	QD core degradation, free radical formation, chromatin condensation, and membrane blebbing	Apoptosis	Hardman (2005), Su et al. (2007), Mahmoudi et al. (2010), Jung and Choi (2006)
Rare earth oxides: Y_2_O_3_, La_2_O_3_, CeO_2_, etc. Mostly 18–60 nm	Lysosomal damage	Pulmonary inflammation and fibrosis	Li et al. (2014)
Gadolinium-based MRI agent: gadodiamide	Nephrogenic systemic fibrosis	Renal failure	Marckmann et al. (2006)
Ag	Decrease in cell survival for endothelial cells and lung cells	Cell death with tissue damage	Korshed et al. (2016)
TiO_2_	Elevated lipid peroxidation	Disruption of cellular respiration	Erdem et al. (2015)
Al_2_O_3_	Disruption of cell membranes	Cell growth inhibition	Ye et al. (2018)
CdO	Endocrine disruption	Death of implanted blastocyst, abnormal foetal dimensions, and diminished growth	Blum et al. (2012)
GO	Immune cell infiltration thickness of 0.93 nm, size of 150–250 nm	Strong inflammation and granuloma formation, cytotoxicity	Yue et al. (2012), Chong et al. (2014), Duch et al. (2011), Ou et al. (2016)

Numerous factors, including the extent of migration and the type of packaging matrix used, may influence NP toxicity (Cushen et al. 2012). Ingesting NPs poses growing health risks, as meals containing NPs may be harmful due to high consumption, bioaccumulation, and overactivity, in addition to the associated hazards (Rasmussen et al. 2010). The toxicity of NPs depends on their type, concentration, duration of exposure, and the sensitivity of the individual (Dimitrijevic et al. 2015).

Beyond concentration and mass, other factors influencing NP toxicity include size, quantity, surface reactivity, surface modification, and aggregation (Muthukrishnan 2022). For instance, smaller silver NPs (10 nm), because of their greater surface area and reactivity, have been shown to enter cells more readily and cause greater cytotoxicity than larger ones (50 nm). Surface modifications, such as PEG coating, can reduce NP toxicity by preventing protein corona formation and enhancing biocompatibility. Aggregation of NPs can also alter toxic effects by changing their biodistribution and lowering cellular uptake. Collectively, these physico-chemical characteristics determine how NPs interact with biological systems and influence their potential risks (AshaRani et al. 2009). NPs can enter the human circulatory system through ingestion into the gastrointestinal tract (GIT) or through inhalation (intranasal or intratracheal), leading to significant deviations from normal physiological functions.

The initial impacts of NPs on the cardiac system include elevated blood pressure, lowered heart rate, and altered vascular tone and dysfunction (Yu et al. 2016). In animal models, for example, inhalation of TiO_2_ NPs has been shown to reduce vascular function and increase arterial pressure (LeBlanc et al. 2009).

When NPs accumulate in various brain regions and alter the expression of genes critical for the development and normal functioning of the central nervous system, they induce cytotoxic effects on neural cells. Accumulated NPs, such as ZnO or silver NPs, can cross the blood-brain barrier and modify the expression of genes related to neurodevelopment and synaptic signaling, ultimately leading to cytotoxicity and neuronal damage (Tang et al. 2009). ZnO NPs have been reported to decrease cell viability, induce apoptosis, alter the cell cycle, and cause oxidative deoxyribonucleic acid (DNA) damage (Yang et al. 2010; Valdiglesias et al. 2013).

A study demonstrated that administering 150 μg/kg of iron oxide NPs to male rats created an imbalance by increasing triiodothyronine (T_3_) hormone levels while decreasing thyroid-stimulating hormone (TSH). Furthermore, palladium NPs have recently been shown to act on hormone receptors, initiating overstimulation and terminating signaling cascades (Jiang et al. 2019; Leso et al. 2018). Excessive NP accumulation has also been identified as one of the main contributors to the present spike in cases of infertility. According to an investigation animal on a high-fat diet, when administered silica NPs which are frequently encountered around workplaces decreased sperm levels and motility rates and elevated sperm abnormality rates (Zhang et al. 2020). TiO_2_ NPs have been found to induce inflammation and cytotoxicity after passing the blood-testis barrier, resulting in genotoxic effects and a substantial loss of sperm DNA integrity (Santonastaso et al. 2019).

NPs frequently aggregate in the proximal convoluted tubules (PCT), where endocytosis may cause tubular cells to internalize the particles after glomerular filtration. A study on T helper type 1 (Th1) cells exposed to inorganic NPs reported DNA damage, and NP-induced nephrotoxicity was also observed (Sramkova et al. 2019). NPs can damage and destroy segments of single-stranded and double-stranded DNA, leading to genetic mutations that manifest as lung cancer and other neoplasms (Sonwani et al. 2021). The introduction, distribution, and absorption of NPs in human systems are directly linked to genotoxicity and cytotoxicity (Stark 2011).

The physicochemical characteristics of NPs – such as their biodistribution, bioavailability, concentration in food products, and levels of consumption – dictate their harmful effects on human organs (Wani and Kothari 2018). Despite their many benefits, nanotechnology in food may negatively affect the environment, society, and human health, as these particles can enter eco-systems through agricultural pesticide use or the processed food industry, including packaging. This raises concerns regarding their potential toxicity (Kalpana Sastry et al. 2013).

## Bioavailability and accumulation

Numerous investigations have explored the use of NMs as delivery systems to improve the bioavailability of bioactive ingredients in dietary supplements (Oehlke et al. 2014). Enhancing bioaccessibility and absorption, as well as modifying any molecular structural changes that may occur during digestion, are strategies for maximizing the bioavailability of bioactive substances. Particle size variation can increase the surface area-to-volume ratio, improving solubility and enhancing bioaccessibility. For instance, because coenzyme Q10 (CoQ10) is poorly soluble in water and lipophilic, its bioavailability is relatively low (Kommuru et al. 2001). Using nanotechnology, foods of exceptional quality can be produced in a more practical way, increasing the bioavailability of nutrients (Dasgupta et al. 2015).

NMs based on trace elements (TEs) also have potential as bioactive agents. According to a report by the World Health Organization (WHO), 21 TEs are essential for sustaining the body’s metabolism and functionality, including selenium (Se), iodine (I), molybdenum (Mo), iron (Fe), copper (Cu), zinc (Zn), chromium (Cr), cobalt (Co), and manganese (Mn). These elements participate in fundamental biological processes as components of proteins, enzymes, and cofactors. Examples include antioxidant defense (e.g., Se in glutathione peroxidase), oxygen transport (e.g., Fe in hemoglobin), and hormone synthesis (e.g., I in thyroid hormones). Mo acts as a cofactor for enzymes such as sulfite oxidase, Cu plays a role in redox reactions and mitochondrial respiration, Zn is involved in over 300 enzymes including DNA polymerase, Cr enhances insulin sensitivity, Co is a component of vitamin B_12_, and Mn contributes to enzyme activation and bone formation. At the nanoscale, these elements often show enhanced bioavailability and targeted interactions, making them promising for medical and nutritional applications.

Currently, four potential pathways of NM metabolism have been identified: elimination via the hepatobiliary and renal systems, interception by the mononuclear phagocyte system (MPS), biodegradation and utilization in the liver, and eventual excretion from the body following gradual breakdown (Cao and Chen 2022). The small size of nanoemulsions also contributes to an extensive surface area, enabling significant interaction with bioactive compounds absorbed in the digestive system. Additionally, nanoemulsions provide more binding sites for digestive enzymes such as lipase and amylase in the intestinal tract, further increasing bioavailability (Gasa-Falcon et al. 2020).

Furthermore, it has been demonstrated that food and humans can bioaccumulate NMs such as nanosilver originating from nanopackaging or from plants and animals (Jovanović 2015). When NPs are exposed to plant or animal tissues, they are more likely to accumulate and persist in those tissues, potentially leading to adverse effects (Speranza et al. 2013). The heightened risk associated with nanoengineered particles arises from their stronger reactivity and the greater bioavailability of smaller particles in the human body, which may cause long-term pathological effects.

By being directly incorporated into novel foods in the form of nanoemulsions, nanocapsules, and nano-antimicrobial films, NMs can enter the food chain. They may also be introduced through nanolaminates and nano-sensors used in food production, processing, preservation, and monitoring. Human exposure to NPs varies considerably depending on the specific application and concentration in the food industry, with the highest risk occurring when NMs are directly applied to food products as carriers of novel ingredients. Ongoing research continues to investigate how food packaging materials contribute to NP migration and how these particles behave once inside the body (Magnuson et al. 2011).

## Chronic stage effects

Nanotoxicity can cause DNA damage, apoptosis, cytotoxicity, uncontrolled cell stimulation, alterations in cell motility, and the development of cancer (Fu et al. 2014). One way to assess the possible risks associated with nanostructured materials is to examine the invasion site, deposition, and migration of NMs throughout the body (Chau et al. 2007). Humans can experience NP accumulation in the kidneys, stomach, lungs, liver, spleen, small intestine, and other major organs of distribution. Moreover, a single oral dose of ZnO NPs can cause complications such as lung damage, kidney disorders, and hepatic injury. The GIT provides a pathway for NP ingestion, as the particles can easily cross biological barriers and enter the circulatory system (Esmaeillou et al. 2013).

NMs also have the potential to cause significant structural damage to mitochondria and DNA mutations, which may result in cell death (Qiao et al. 2024). Carbon nanotubes, commonly used in food packaging, are hazardous to human skin and lungs (Mills and Hazafy 2009). The deliberate integration of intelligent and active food packaging components, compared with conventional packaging materials, presents new challenges for safety assessment. The primary concern with food contact materials (FCMs) is the migration of potentially harmful substances from packaging into food at levels exceeding established safety limits (Dainelli et al. 2008). NMs also interact with the immune system, with the ability to stimulate or, in some cases, suppress immune responses (Boraschi et al. 2017).

## Environmental impact

NPs can enter the body through cutaneous contact, ingestion, or inhalation. Significant concern arises from the large quantities of NMs used in food packaging because of their potential release into the environment or into contaminated food (Han et al. 2018). As the use of nanoproducts increases, concerns regarding environmental and human health are also growing due to the unique physicochemical properties of NMs (He et al. 2018). Public concern continues to rise about the potential toxicity of NPs in biological systems. Current research is focusing on the possible harm NMs could cause as a new source of environmental contaminants (Moore 2006). Chemically produced NMs are not environmentally friendly, as they require a long time to completely degrade. Consequently, the dose and degree of environmental exposure to nanoproducts determine the extent of toxicity to living systems (Yadav et al. 2023). Once discharged, NPs are almost impossible to retrieve. Airborne NPs can rapidly infiltrate groundwater and soil, eventually spreading into vegetation, crops, and the water cycle (Mitter and Hussey 2019). The use of NPs as nanopesticides, nanoherbicides, nanofertilizers, and, less commonly, immobilized nanosensors has raised concerns about environmental health. Once absorbed, NPs may become potentially hazardous to plants directly or indirectly through the release of toxic ions during NP disintegration. Reported effects include reduced germination, biomass, and root and leaf growth (Shen et al. 2010; Hong et al. 2014). For example, changes in photosynthetic indices were observed in cucumbers exposed to 200 mg/l of CeO_2_ and copper peroxide (CuO_2_) NPs (Hong et al. 2016). The release of NMs into air, water, or soil could therefore have harmful environmental consequences (Cardoza et al. 2022).

## Emerging technologies for food safety and quality

With its numerous applications in food processing, security, and safety, as well as in enhancing nutraceutical value, prolonging shelf life, and reducing packaging waste, recent advances in nanotechnology have significantly transformed the food industry (Wesley et al. 2014). The food sector applies nanotechnology in many different ways. Table 3 illustrates the diverse applications of nanotechnology in food systems.

**Table 3:** Diverse applications of nanotechnology in food systems

S. no.	Nanomaterial	Food product	Applications
1	Nanosized self-assembled liquid structures (NSSL)	Food and beverage	Inhibits the transportation of cholesterol from the digestive system into the bloodstream
2	Nanoselenium	Beverage	Good supplement of selenium
3	Micelles 5–100 nm in diameter	Health drink	Increased lycopene
4	Conversion of vanilla or chocolate into nanoscale	Health drink	Low-calorie diet
5	Liquid-suspended nanoparticle	Food and beverage	Low-calorie diet
6	Nanosized self-assembled liquid structures	Food	Nanocapsules of omega-3 fatty acids
7	Nanodroplets	Food supplements	Efficiency enhancement
8	Silicon	Health supplement	Health and fitness
9	300 nm of iron particles	Beverage	Increases reactivity and bioavailability
10	Nanoscale micelle	Food additive	Increases absorption and effectiveness of nutritional additives and preservatives
11	Nanocochleates as small as 50 nm	Food additive	Effective addition of omega-3 fatty acids
12	< 200 nm synthetic lycopene	Food additive	Potent antioxidant and used in soft drinks
13	Nanoparticles of silver	Food contact material	Potent antibacterial
14	25 nm of silver nanoparticles	Food storage	Antimicrobial protection
15	Plastic	Food storage	Longevity of food products
16	Nanosilver	Food storage	Strong disinfection and storage power
17	Silver	Food storage	Food storage
18	Nanomicelle	Sustain beverage	Introduce antioxidants into food and beverage products
19	Nanocolloidal silicate mineral and Hydracel^®^	Nanosized powders	Neutralize free radicals, lower the surface tension of drinking water, and increase solvent properties
20	Liposomal nanospheres	Supplements	Health application
21	Silver nanoparticle	Fortified Jambu Juice	Rich in 22 essential vitamins and minerals
22	Silver NPs	Supplemented functional drink	Antibacterial and antifungal effects as a surface disinfectant
23	Silver hydrosols	Supplemented functional drink	Sterilization and quality control
24	Silver NPs	Supplemented functional drink	Antibacterial activity and sterilization effect
25	Silver NPs	Supplemented functional drink	Highest bioavailability
26	Silver NPs	Supplemented functional drink	Supports the immune system and defence for natural healing
27	Colloidal silver	Supplemented functional drink	Sterilization
28	Colloidal silver consists of small nanoparticles of metallic silver	Food supplement	Colloidal silver particles can be excreted
29	Actively charged nanocolloidal silver hydrosol	Food supplement	Safely supports the immune system
30	Silver	Food supplement	Support natural healing

Food and beverage manufacturers can efficiently incorporate β-carotene using nanoemulsions (Mehmood et al. 2021). Nanonutraceuticals are developed through nanoformulation techniques to produce functional foods, bioactive compounds, vitamin and mineral supplements, and herbal products. Numerous delivery systems – such as liposomes, cubosomes, microemulsions, single-layered structures, biopolymeric NPs, microgels, and fibers – are employed to transport nutraceuticals via nanotubes, nanofibers, fullerenes, nanosheets, and nanowhiskers (He et al. 2019; Nile et al. 2020).

Nanotechnology has also been applied in smart distribution, packaging, and protection (Chen and Yada 2011). Several types of biosensing tools are employed in the food sector, including those for bioscience research, environmental research, and applications involving graphene, reduced graphene oxide, and graphene-based graphene oxide (Taniselass et al. 2019). Packaging applications are often antimicrobial in nature, serving as carriers of antimicrobial polypeptides and protecting against microbial degradation. Bacterial growth can be inhibited by packaging materials consisting of starch colloidal outer shells loaded with antimicrobial agents, which release controlled amounts into the packaged product.

Nanofilters are used to extract lactose from milk and replace it with other sugars, making it suitable for individuals with lactose intolerance. NM-based nanosieve filters are also employed to eliminate bacteria and filter milk, beer, and water (Agriopoulou et al. 2020; Nile et al. 2020). Edible nanocoatings, with a thickness of around 5 nm, act as gas and moisture barriers in baked goods, fast foods, cheeses, fruits, and vegetables. Multiple bakery products on the market already use edible nanocoated antimicrobials. These coatings may include nanostructured gelling agents such as gelatin NPs, cellulose nanocrystals, chitosan films with nano-silicon dioxide (SiO_2_), nanosilica-chitosan coatings, and nanolaminate coatings composed of lysozyme and alginate. Such technologies have been applied to preserve fresh foods for extended periods (Singh et al. 2017).

Recent research by Thuong et al. (2020) demonstrated that incorporating silica filler into natural rubber composites significantly enhances their mechanical properties. Tensile strength increased sevenfold, and loss modulus increased twenty-fivefold. Nanotechnologies in food packaging materials with integrated nano-sensors can monitor alterations in food processing, whether chemical, biological, or physical (Onyeaka et al. 2022; Pathakoti et al. 2017).

The discharge of metal ions at the surface, from within, or across the cell can alter cellular structure or function (Krzywoszyńska et al. 2020). Recent advances in the targeted delivery of Clustered Regularly Interspaced Short Palindromic Repeats (CRISPR)/CRISPR-associated (Cas) proteins, messenger ribonucleic acid (mRNA), and single-guide RNA (sgRNA) for crop genome editing – leveraging tissue engineering and engineered NMs – represent a remarkable scientific achievement in nanofarming (Kim et al., 2017; Shang et al., 2019). Nanoformulations containing pesticides have also demonstrated improved controlled release, enhanced efficacy, and higher crop yields (Petosa et al. 2017; Ul Haq and Ijaz 2019).

## Risk assessment and safety evaluation

Due to their very small size, NPs have the ability to pass through cell membranes and cause genotoxicity (Fajardo et al. 2022). Assessing the degree of exposure to NPs is crucial to determine the type and extent of damage these microscopic particles may cause to different kinds of viable cells and tissues. The three main exposure routes for NPs are through the skin, the nose and throat, and the gastrointestinal system (Sahu and Hayes 2017).

Exposure of cells to certain NMs has been associated with DNA damage, which may result in interstand and intrastand breaks, single-strand and double-strand breaks, and genomic rearrangements. In addition, it has been shown that transformed bases such as 5-hydroxy-5-methylhydantoin, thymine glycol, and 8-hydroxyguanine can form (Biola-Clier et al. 2017). Regulation of the risks associated with nanofood and the adoption of nanotechnologies in the food manufacturing industry are of paramount importance. Broader civil rights, social, economic, and ethical concerns raised by nanotechnology must also be addressed by federal and state governments. For democracies to maintain control over these technological advancements in food and agriculture, civic engagement in nanotechnology governance is considered essential (Sodano et al. 2016). To assess public opinion and perception of NPs in Switzerland, a convenience sample survey was conducted from August to October 2020 using participants from the Adolphe Merkle Institute and the University of Fribourg’s email list. The study revealed that a significant percentage of participants had very limited knowledge about NPs unintentionally incorporated into food products (Rothen-Rutishauser et al. 2021). According to the WHO, the EU, the United Nations (UN), and the Food and Agriculture Organization (FAO), the safety of NPs in food must be further strengthened (Magnuson et al. 2013).

Nanotechnology can extend the shelf life of food packaging and prevent nutrient loss and degradation, thereby ensuring food safety (Graveland-Bikker and de Kruif 2006). Because there is always an unknown risk associated with the use of NMs as pesticides, microbicides, and activation catalysts, risk assessment methods must be strictly followed in food processing (Shi et al. 2013). In *in vivo* toxicological studies using mammalian models such as mice and rats, the quantity of silver NPs tested remains very small (Mao et al. 2016). Public concern continues to grow regarding the possible toxicity of NPs in biological systems. NMs have the potential to represent a new form of environmental pollution, and current research is focusing on determining their potential negative impacts (Shah and Mraz 2019). Guidelines for evaluating the risks associated with the application of nanoscience and nanotechnology in the food and feed chain have been published by the European Food Safety Authority (EFSA) (Hardy et al. 2018).

## Labeling requirements for nanotechnology in food

The use of NPs has also enabled the development of “smart” and “intelligent” labeling concepts, as well as functional food contact materials (FCMs) that are more resilient, lightweight, and practical. Conventional food packaging is inert by design, whereas intelligent and active FCMs are specifically designed to interact with food, extending shelf life by preserving or enhancing the condition of packaged products. This can be achieved by transferring or absorbing substances into or out of the food and its environment, such as through a disinfectant agent, or by using a labeling system that indicates food expiry – for example, by changing color when the maximum storage temperature or shelf life is exceeded (a process known as freshness monitoring). Moreover, according to labeling requirements, the term “nano” must always follow the component name on labels containing NMs (Gottardo et al. 2021).

## Consumer awareness and perception

Two guidance documents have been released recently by the EFSA. To ensure consumer protection, one outlines the technical requirements for demonstrating the presence of minute particles or determining whether a product’s nanoscale properties persist during use. The other guides scientific risk assessment and appropriate safety testing of NMs (Schoonjans et al. 2023). Although public acceptance and awareness play a significant role, food manufacturers often overlook them. In fact, many manufacturers prefer to develop new products “underground” and keep them hidden from the public, possibly due to competition and trade secrecy (Chun 2009). A case study in Singapore showed that the public’s negative perception of nanotechnology is worsened by limited awareness of its harmful implications (George et al. 2014). Businesses should also take note of food product policies that incorporate packaging technologies intended to benefit consumers. While some view such packaging as a useful tool, others regard it as a strategy to promote sales (Siddiqui et al. 2022). To effectively communicate with consumers and influence other stakeholders – such as government bodies regulating NMs in food packaging and industries applying nanotechnology in food systems – the media and publishers play a crucial role (Bumbudsanpharoke and Ko 2015).

The dual public voices about nanotechnology in the food industry are assent and altruism (Brown et al. 2015). Consumer food preferences are complex, influenced not only by economic and health concerns but also by social and psychological factors. However, studies on consumer behavior have typically focused on economic and health-related aspects, with social and psychological dimensions receiving less attention (Huang et al. 2020). Today, packaging design is significant because consumers expect it to reflect their aspirations for health and well-being. Packaging can also influence consumption habits and promote healthier lifestyles as a marketing tool (Bou-Mitri et al. 2021). A major challenge for the food industry is meeting consumer demand for food that is safer, more convenient, higher quality, and more natural. The demand for environmentally friendly packaging and products, produced through sustainable and efficient processes, is steadily increasing (Trajkovska Petkoska et al. 2021). Likewise, the need for smart foods with enhanced nutritional value is rising due to changing dietary habits and the rapid pace of urbanization (Nayak et al. 2021).

## Future directions

The majority of nations producing NMs lack appropriate laws about nanotechnology. Therefore, comprehensive legislation and regulations, along with stringent toxicological screening procedures, are necessary for the permissible use of nanotechnology (Neme et al. 2021). A clearly defined regulatory objective is also required for the effective control of nanotechnologies in the food industry (Fletcher and Bartholomaeus 2011). Future progress lies with researchers in developing more efficient and adequate nanocarriers with enhanced bioavailability that preserve food’s flavor, quality, and appearance during carrier incorporation. When antigen-specific markers are used to create polymer nanocomposite films by incorporating NPs into food packaging, the concept of smart packaging can be fully realized (Hamad et al. 2018). Nanotechnology is also valuable in plant disease prevention and agricultural development (Tripathi et al. 2017). Biosafety regulations are essential for the safe application of synthetic, eco-friendly NPs in the food and agricultural sectors. For example, the Food and Drug Administration (FDA) in the United States regulates food packaging and nano-enabled foods, while the European Union oversees food additives developed using nanotechnology (Gupta et al. 2023).

The next wave of the agri-tech revolution will heavily rely on nanotechnological interventions. Despite its potential, nanotechnology faces several challenges. It has given rise to a number of tools for enhancing the agronomic traits of plants, including those for improving stress tolerance, increasing plant resistance to fertilizers and pesticides, and developing nanosensors for smart agriculture and plant genetic engineering (Kumari et al. 2023). Future food packaging materials could also be transformed by carbon nanotubes, potentially leading to active and intelligent packaging systems (Wang and Irudayaraj 2008).

The global forum for discussing nanosafety issues is the UN Strategic Approach to International Chemicals Management (SAICM). SAICM incorporated new activities related to NMs and nanotechnologies into its Global Plan of Action, along with a nano-specific resolution (Karlaganis et al. 2019).

With its ability to manipulate matter at the atomic level, nanotechnology holds considerable potential to revolutionize many aspects of medical care, including drug delivery, regenerative medicine, equipment operation, diagnosis, and disease monitoring. It also provides access to advanced research tools that can aid in the development of medications for various conditions. Nanotechnology can be used to deliver drugs to specific body cells, thereby reducing the likelihood of failure or rejection (Haleem et al. 2023). Applications of nano-technology have enabled earlier disease identification, including the use of carbon nanotubes, Au nanorods, and rapid, cost-effective detection methods. Smart tablets equipped with nanobots designed to target specific cancer cells could be used to diagnose and ensure that affected individuals receive treatment as directed. Tools and processes also improve the safety, efficacy, and physiochemical characterization evaluations of NMs and nanosurfaces in medical device engineering (Haleem et al. 2021; Zhang et al. 2021). NanoFlares are particles engineered to bind to specific genetic targets on cancer cells and illuminate upon detection. Cancer nanomedicine is a relatively new field of study, and regenerative immune sensors represent an emerging area of interest, allowing semicontinuous monitoring and recurring patterns for statistical reliability (Wang et al. 2021; Jurj et al. 2017; Sarmah et al. 2021; Rae and Jachimska 2021).

## Conclusions

This review highlights the role of nanotechnology in the food system, summarizing its opportunities and risks for human health while exploring the types of NMs used in food, their regulatory approaches, safety evaluations, and risk assessments. The integration of nanotechnology in food science offers numerous benefits, including improved food quality, enhanced safety, and greater sustainability, and its applications are expected to expand significantly. NPs pave the way for innovation, addressing complex and persistent challenges within the food industry. The incorporation of advanced nanotechnologies is projected to have a transformative impact on food systems, a crucial element of human nutrition. Staying informed about emerging technologies is essential to fully leverage their potential in food science.

Nanotechnology provides innovative solutions to key challenges, such as improving safety, enhancing nutrition, reducing waste, and promoting sustainability, while meeting the rising demand for high-quality, health-focused products. However, concerns about its potential toxicity must be addressed. NPs may accumulate in the body, potentially causing cellular damage, oxidative stress, or inflammation. Moreover, the long-term effects of NP ingestion on human health and the environment remain unclear. Rigorous safety evaluations, regulatory frameworks, and responsible implementation are critical to mitigating these risks.

Despite these challenges, nanotechnology offers a promising path forward, with the potential to provide targeted therapies, improve treatment efficacy, and reduce environmental impact. Ongoing research is essential to fully understand the behavior of NMs and their effects on human health, ensuring a safer and more sustainable future for the food industry. Future research in food nanotechnology should prioritize safety, sustainability, and long-term impacts. Policies must ensure clear labeling, standardized testing, and transparent risk assessments to build consumer trust.
